# A deep dive into shrimp allergy: clinical spectrum of shrimp allergy in a Tunisian pilot study

**DOI:** 10.3389/falgy.2025.1568475

**Published:** 2025-04-30

**Authors:** Dhouha Krir, Imen Zamali, Yousr Galai, Ahlem Ben Hmid, Ines Ben Sghaier, Yosra Nasri, Hayet Kebaier, Hechmi Louzir, Nissaf Ben Alaya Bouafif, Mélika Ben Ahmed, Samar Samoud

**Affiliations:** ^1^ Laboratory of Clinical Immunology, Institut Pasteur de Tunis, Tunis, Tunisia; ^2^ Faculty of Medicine of Tunis, University of Tunis El Manar, Tunis, Tunisia; ^3^ Laboratory of Transmission, Control and Immunobiology of Infection, Institut Pasteur de Tunis, Tunis, Tunisia; ^4^ Faculty of Pharmacy of Monastir, University of Monastir, Monastir, Tunisia; ^5^ National Observatory of New and Emerging Diseases, Ministry of Health, Tunis, Tunisia; ^6^ Faculty of Medicine of Sousse, University of Sousse, Sousse, Tunisia

**Keywords:** shrimp allergy, molecular allergen, specific IgE, cross-reactivity, tropomyosin

## Abstract

Shrimp allergy has emerged as a growing health concern in Tunisia, likely due to changing dietary habits. This study aimed to characterize the clinical features of shrimp-allergic patients and investigate potential cross-reactivity with house dust mites (HDMs) and snails using *in vitro* diagnostic methods. Thirty-one patients with a self-reported history of shrimp allergy were referred to the Clinical Immunology Department of the Pasteur Institute of Tunis. Total IgE and Serum-specific IgE (sIgE) levels to shrimp, snail, and HDMs, as well as recombinant allergens rPen a1 and rDer p10, were measured using the ImmunoCAP® immunoassay. The study population consisted mainly of young adults [mean age: 15.5 years (10–27.2)], with a male-to-female ratio of 1.4. The most common symptoms were oropharyngeal pruritus and urticaria. Shrimp allergy was confirmed in 54.8% of patients, with a median sIgE titer of 0.18 [0.03–28.8] kUA/L. Among these patients, 58.8% exhibited cross-reactivity, predominantly with snails [median sIgE: 3.07 (0.04–16.85) kUA/L]. Among shrimp-allergic patients, 70.5% tested positive for rPen a1 [median sIgE: 28.42 (5.78–51.05) kUA/L], while 58.8% were positive for rDer p10 (median sIgE: 0.56 [5 × 10^−5^–87.95] kUA/L). The median total IgE level was 297 [158.6–475] IU/ml, significantly higher in shrimp-allergic patients (*p* = 0.005). The median shrimp sIgE/total IgE ratio was 0.001 [0–0.069], also significantly elevated in shrimp-sensitized individuals (*p* = 0.005). Multivariable analysis showed significant correlations between total IgE and shrimp sIgE, rPen a1, and rDer p10 levels (*p* = 0.043, *p* = 0.045, *p* = 0.043, respectively), while no correlation was found with d1 or snail sIgE after adjusting for age. rDer p10 and f24 were the strongest predictors of sIgE to snail, with standardized coefficients of 8.785 and −5.028, respectively. However, these associations did not reach statistical significance. This study underscores the critical role of tropomyosin as a primary allergen in shrimp allergy in Tunisia, highlighting its importance in immunodiagnosis and its strong association with HDMs and snail sensitization. Further research is needed to explore HDMs sensitization in patients who are negative for rPen a1 and rDer p10.

## 1 Introduction

Crustaceans' allergy, with shrimp being a leading culpit, is one of the most prevalent food hypersensitivities worldwide, in both children and adults, with significant implications for public health (1, 2). In recent years, significant advances in molecular diagnosis have enabled the characterization, clonig and recombinant production of allergenic major and minor components and epitope-emulating peptides from vaious shellfish species to be employed for the quantification of specific IgE antibodies (sIgE). Tropomyosin (Pen m1, Pen a1) was the first major shrimp allergen indentified in the early 1980 (3, 4). It is a 37 kDa heat-resistant pan-allergen (5) and boiling might enhance their allergenicity by exposing neo-epitopes (6–8). Isoforms have been identified in several shrimp species sharing a sequence identity between 91% and 100%. The first recombinant tropomyosin synthesized, rPen a1, was obtained from Penaeus aztecushas and has become a superior diagnostic tool with enhanced specificity in comparaison with whole-shrimp extracts (9, 10). Moreover, tropomyosin from house dust mites (HDMs) and cockroaches share high amino-acid sequence homology (78.5%—81.7% and 82.4% respectively) to shrimp tropomyosin rPen a1 (11). Cross-reactivity between tropomyosins from HDMs and shellfish has been proven (12) and might be the key for a better understanding and an optimized diagnosis of shrimp allergy. This study represents the first comprehensive analysis of the phenotypic profile of shrimp allergy in Tunisia, examining its cross-reactivity with house dust mites and snails, primarily driven by sensitization to tropomyosin.

## 2 Methods

### 2.1 Patients and study design

This was a retrospective analytical study conducted at the Clinical Immunology Department of the Pasteur Institute of Tunis, including a group of patients suspected of having a shrimp allergy, consulting at our national reference laboratory covering all the country. A total of 31 patients were retrospectively recruited for this study between January 1, 2021, and October 1, 2024. Referrals were received from various specialists, including pulmonologist-allergist, and pediatricians covering the different region of Tunisia. Clinical *data* for these patients were retrieved from their respective referring centers. Patient demographics, including age, sex, allergy history, associated allergies, and clinical manifestations of shrimp allergy, were collected at baseline and at the final follow-up if available.

One of the following criteria was required for inclusion in the study:

- Documented history of immediate allergic reactions within shrimp consumption.
- Self-reported severe shrimp disgust.

Patients with incidental detection of shrimp-specific IgE by semi-quantitative immunoblot assay in the absence of a clinical history of shrimp allergy were excluded. Additionally, patients with missing data were excluded from the analysis.

Additional specific IgE tests for associated food and respiratory allergies were performed on a case-by-case basis at the clinician's request, following suspicion of cross-reactivity or associated allergy to shrimp, or in the presence of positive skin prick test results.

Skin prick testing and prick-to-prick testing for shrimp were not performed on any patient.

### 2.2 IgE analysis

Blood samples were collected from participants into evacuated tubes without anticoagulant. After centrifugation at 3,500 ×  g at room temperature, the sera were aliquoted and stored at −20°C. Shrimp-specific IgE (sIgE) levels were then measured for *in vitro* diagnosis using the Phadia™ 100 automated system (Thermo Fisher Scientific®, Uppsala, Sweden) with the ImmunoCAP® fluoroimmunoassay for shrimp (f24). Sera from patients with positive shrimp sIgE levels were further analyzed for sIgE to recombinant shrimp tropomyosin (rPen a 1) using the ImmunoCAP® assay. Patients with positive shrimp sIgE levels underwent screening for sIgE antibodies against the major HDMs allergen *Dermatophagoïdes pteronyssinus* (d1), using the ImmunoCAP® assay. Participants with positive d1 results were further tested for IgE specific to recombinant Der p10 (rDer p10) using the same assay. This subgroup also underwent additional screening for sIgE to snails to explore potential cross-reactive allergies associated with shrimp allergy. A cut-off value of 0.1 kUA/L was used to define positive ImmunoCAP® sIgE test results, following established guidelines (13). The assay has an upper limit of quantification (ULOQ) of 100 kUA/L.

Total serum IgE levels (tIgE) were measured in all patients included in the study. Test results were interpreted according to age-specific reference values to determine positivity and were expressed in IU/ml (14, 15). Subsequently, the ratios of shrimp sIgE to tIgE and rPen a1 to tIgE were calculated.

### 2.3 Statistics

Statistical analysis was performed using the IBM Statistical Package for the Social Sciences (SPSS) version 11 (IBM®, Armonk, USA) and GraphPad Prism 5 (Dotmatics®). Our results were expressed as percentages for categorical variables. The distribution of the variables was assessed for normality using skewness, kurtosis, and Shapiro–Wilk tests. For variables exhibiting a high degree of skewness, we reported median values and interquartile ranges. We expressed our results as the mean and standard deviation for variables with a Gaussian distribution. To assess the relationship between quantitative variables, Pearson's correlation coefficient and Spearman's rank correlation coefficient (r_s) were employed, with the choice of test guided by the data distribution and the linearity of the relationship. Additionally, the *F*-test was applied in the context of regression analysis to evaluate the significance of Spearman's rank correlation. Receiver operating characteristic (ROC) curves were used for: the comparison of sIgE f24 and rPen a1 levels, for the discriminatory ability of IgE levels of rPen a1 to predict rDer p10 sensitization and for the discriminatory ability of IgE levels of rPen a1 to predict sensitization to snail. For the multivariable analysis to study explanatory variables associated with the main outcomes, total IgE, shrimps sIgE and snail sensitization, we applied multiple linear regression. We considered the final model with the significant variables adjusted for age. For all statistical analyses, a significance level of 0.05 was adopted, indicating a maximum acceptable type I error rate of 5%.

### 2.4 Ethics

The study protocol, including procedures for informed consent, was reviewed and approved by the Ethics Committee of the Faculty of Medicine of Sousse (CEFMSo_0002_2025). All clinical data were collected with informed consent from participants and their treating physicians. All data were anonymized prior to analysis.

## 3 Results

### 3.1 Patient demographics, clinical and biological characteristics

**Figure 1:**
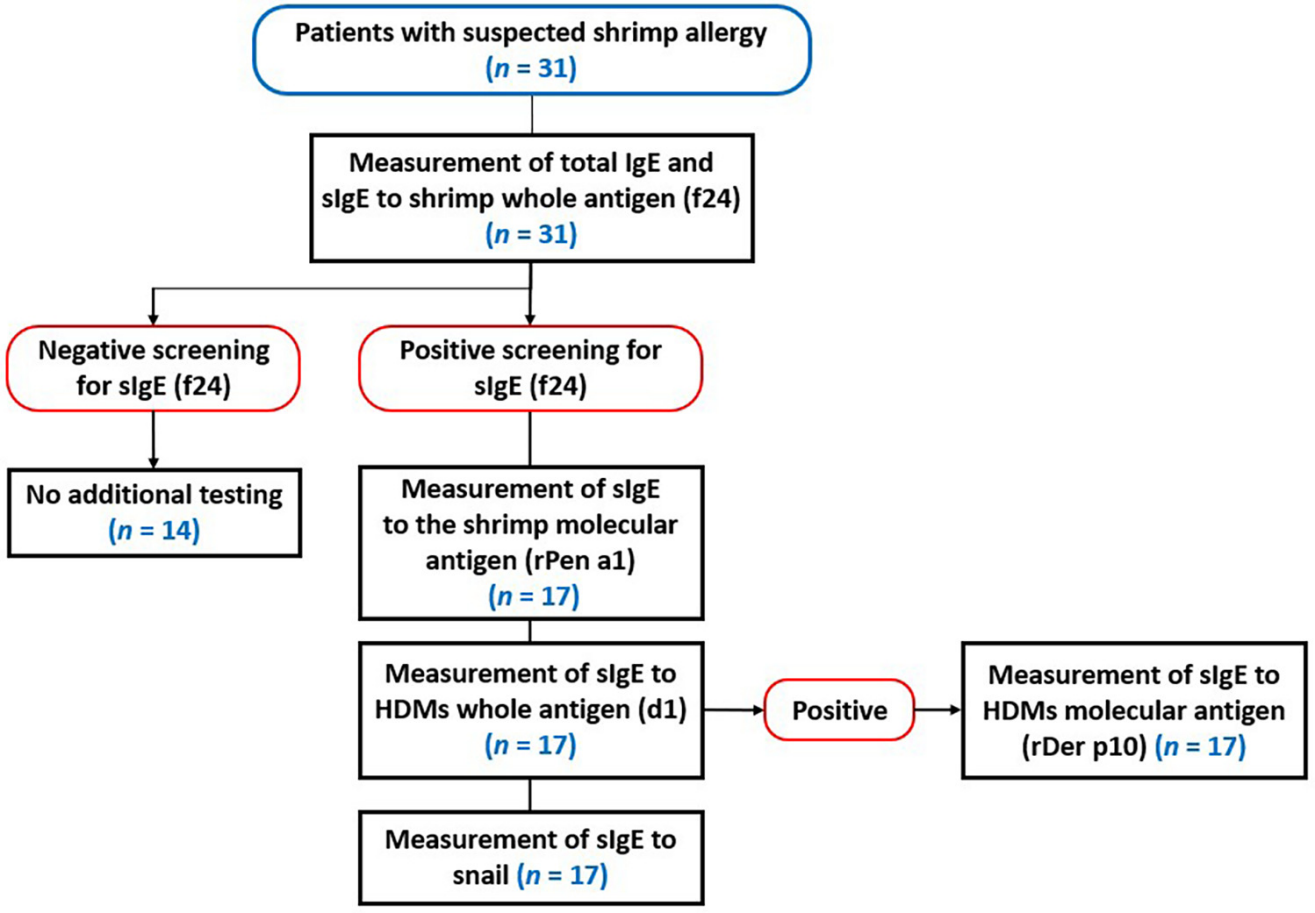
Flowchart for diagnosing shrimp allergy and assessing potential cross-reactivity with HDMs and snails.

Thirty-one patients with clinical suspected shrimp allergy were referred to our laboratory for potential study participation by various specialists, including pulmonologist-allergist (*n* = 21) and pediatricians (*n* = 10). The study population comprised 18 males and 13 females [sex-ratio (male/female) of 1.4], predominantly consisting of young adults with a median age of 15.5 [10–27.2] years. The age distribution of the population was not normal (*p* = 0.002). Patient's immunological measurements were done according to the following algorithm (Figure 1):

### 3.2 Demographics, clinical and biological characteristics of patients sensitized to shrimp

Shrimp-sIgE levels were measured in all 31 enrolled patients. The median titer was 0.18 kUA/L [0.03–28.8] kUA/L, with seventeen patients (54.8%) testing positive. Even though we noticed a tendency for sIgE titer to increase with age, Univariate and multivariable analysis revealed no statistically significant correlation between shrimp-specific IgE (sIgE) titers and age (r_s = 0.210, *p* = 0.303 and *p* = 0.517, respectively). The group of patients sensitized to shrimp included 11 males and 6 females (sex-ratio = 1.8), with predominance of young adults [16 (12–28) years]. The median of the shrimp sIgE titer was 25.9 [1.01–95.00] kUA/L. Upon exposure to shrimp, these patients exhibited a range of allergic reactions. Most prevalent symptoms were oropharyngeal pruritus (*N* = 11, 64.7%) and allergic rhinitis (*N* = 10, 58.8%). Additional reported symptoms included allergic asthma (*N* = 2, 11.6%), shrimp aversion (*N* = 3, 17.6%), urticaria (*N* = 5, 29.4%), anaphylaxis (Grade III/IV) (*N* = 3, 17.6%), rapid edema (*N* = 3, 17.6%), eosinophilic esophagitis (*N* = 1, 5.8%) and diverse gastrointestinal symptoms (*N* = 1, 5.8%). The incidence and severity of these reactions varied among patients (Table 1).

**Table 1:** Clinical characteristics of patients with shrimp-specific IgE positivity.

Patients	Age (years)	Gender	sIgE to f24 (kUA/L)	Oral-pharyngeal pruritus	Asthma/Allergic rhinitis	Urticaria	Shrimp disgust	Anaphylaxis^a^	Eosinophilic esophagitis	Quick edema
Patient 1	27	F	1.91	+					+	
Patient 2	21	F	>100	+						
Patient 3	11	M	90	+						
Patient 4	28	M	3.64	+	+					+
Patient 5	27	M	28.8	+	+					
Patient 6	15	M	>100					+		
Patient 7	12	M	>100		+		+			
Patient 8	28	M	25.9		+					
Patient 9	73	M	3.48	+	+	+		+		+
Patient 10	10	M	48.1	+		+	+			
Patient 11	21	M	>100	+		+				
Patient 12	17	F	89.4	+	+	+		+		
Patient 13	11	M	0.1		+		+			
Patient 14	47	F	0.61	+	+	+				
Patient 14	16	F	1.12							
Patient 16	14	M	0.18	+	+					
Patient 17	15	F	100		+					

^a^ Anaphylaxis grade III or IV.

F: Female; M: Male.

### 3.3 Total IgE levels and associated factors

**Figure 2:**
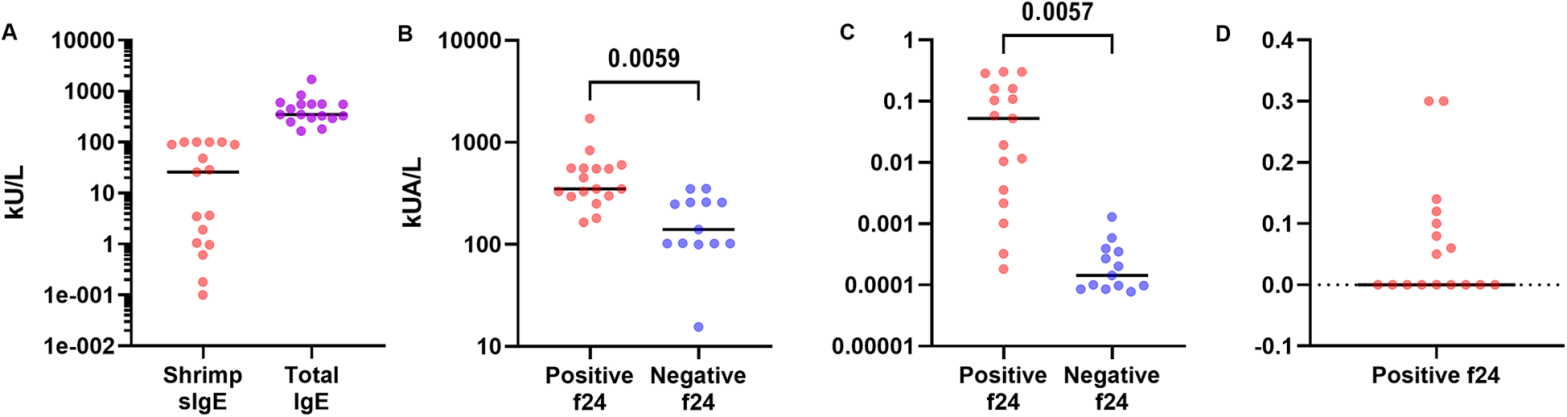
Analysis of total IgE levels. **(A)** Comparison of Total IgE and Shrimp sIgE Levels, **(B)** comparison of total IgE levels by f24 positivity and **(C)** comparison of shrimp sIgE/tIgE levels ratio by f24 positivity **(D)** rPen a1/tIgE levels ratio.

The median tIgE levels measured in all patients (*n* = 31) was 297 [158.6–475] IU/ml. These levels were significantly higher in shrimp allergic patients compared to those who were not (*p* = 0.005) (Figure 2). The median ratio of shrimp sIgE to tIgE was 0.001 [0–0.069]. This ratio was also significantly higher in shrimp-sensitized patients (*p* = 0.005). The median ratio of rPen a1 to tIgE was 0.002 [0–0.109] (Figure 2).

Multivariable analysis demonstrated a statistically significant correlation between tIgE levels and shrimp sIgE (f24), rPen a1 and rDer p10 levels (*p* = 0.043, *p* = 0.045, *p* = 0.043, respectively). No correlation was found between tIgE levels and d1 or snail sIgE, after adjusting for age (Table 2). Results demonstrated a statistically significant correlation between the shrimp sIgE/tIgE ratio and the levels of shrimp sIgE (f24), rPen a1, and rDer p10 (*p* = 0.003, *p* = 0.002, and *p* = 0.004, respectively). However, no correlation was found between this ratio and d1 or snail sIgE after adjusting for age (Table 3). Furthermore, the shrimp sIgE/tIgE ratio was not significantly associated with symptom severity (*p* = 0.694). Similarly, the rPen a1/tIgE ratio showed a significant correlation with shrimp sIgE (f24), rPen a1, and rDer p10 levels (*p* = 0.043, *p* = 0.045, and *p* = 0.043, respectively). In contrast, no correlation was observed between this ratio and d1 or snail sIgE after adjusting for age (Table 3). Additionally, it was not significantly linked to symptom severity (*p* = 0.737).

**Table 2:** Associated factors to tIgE levels and ratios by multivariable analysis.

Dependent variable	Final model	Beta coefficient	*T* test	*p*	CI 95% beta coefficient	
					Inf	sup
Total IgE.	f24	−11.196	−2.697	0.043	−97.730	−2.341
	rPen a1	−8.391	−2.654	0.045	−75.449	−1.209
	d1	0.410	1.639	0.162	−1.673	7.562
	rDer p10	19.903	2.700	0.043	4.226	171.996
	Snail sIgE	−0.400	−0.743	0.491	−30.734	16.949
	Age	−0.282	−1.092	0.325	−9.868	3.983
Shrimp sIgE/tIgE ratio	f24	5.890	5.541	0.003	0.008	0.022
	rPen a1	4.650	5.745	0.002	0.007	0.018
	d1	−0.015	−0.239	0.820	−0.001	0.001
	rDer p10	−9.507	−5.038	0.004	−0.037	−0.012
	Snail sIgE	−0.076	−0.551	0.605	−0.004	0.003
	Age	−0.024	−0.365	0.730	−0.001	0.001
rPen a1/tIgE ratio	f24	5.057	3.732	0.014	0.004	0.022
	rPen a1	5.209	5.048	0.004	0.007	0.021
	d1	−0.004	−0.044	0.967	−0.001	0.001
	rDer p10	−9.225	−3.835	0.012	−0.040	−0.008
	Snail sIgE	−0.105	−0.597	0.576	−0.006	0.003
	Age	−0.048	−0.576	0.590	−0.002	0.001

**Table 3:** Cross-reactive allergies in patients with shrimp sIgE positivity.

Cross-allergies	Frequency	sIgE (kUA/L) mean/median
Respiratory allergies		
House dust mites	16/16	35.71 ± 27.58
Cockroaches	1/1	12.1
Food allergies		
Crab	2/2	1.67
Snail	10/17	3.07 [0.04–16.85]

### 3.4 Predictive factors of sIgE to shrimp

**Figure 3:**
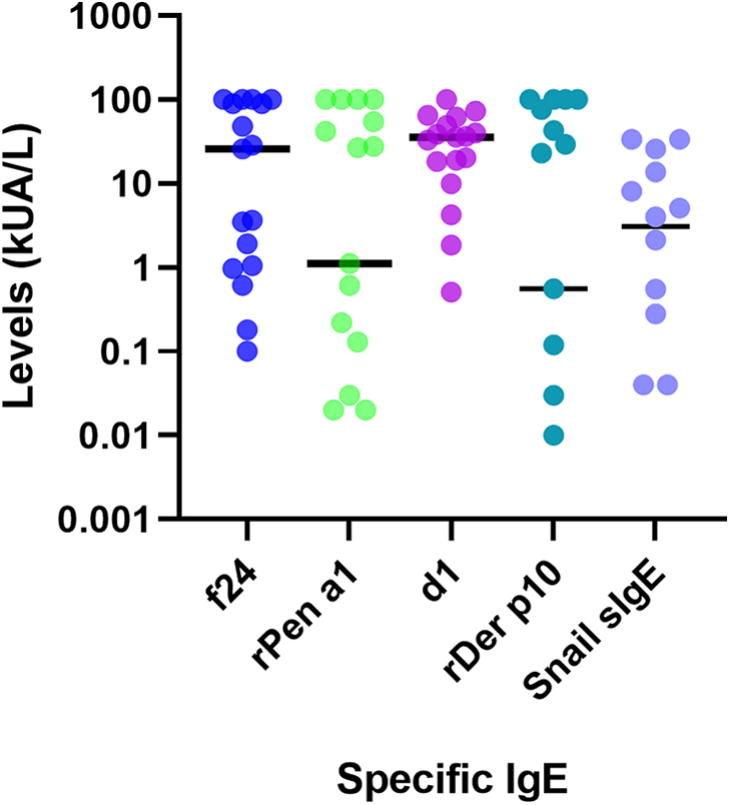
Analysis of sIgE levels to shrimp (f24, rPen a1), HDMs (d1, rDer p10) and snail.

All 17 participants with positive shrimp sIgE levels exhibited sensitization to HDMs allergens, as evidenced by the presence of IgE antibodies against d1. While some patients reported no clinical manifestations, all were confirmed to be sensitized to HDMs allergens. The median titer for d1 was 35.7 ± 27.5 kUA/L (Figure 3).

**Figure 4:**
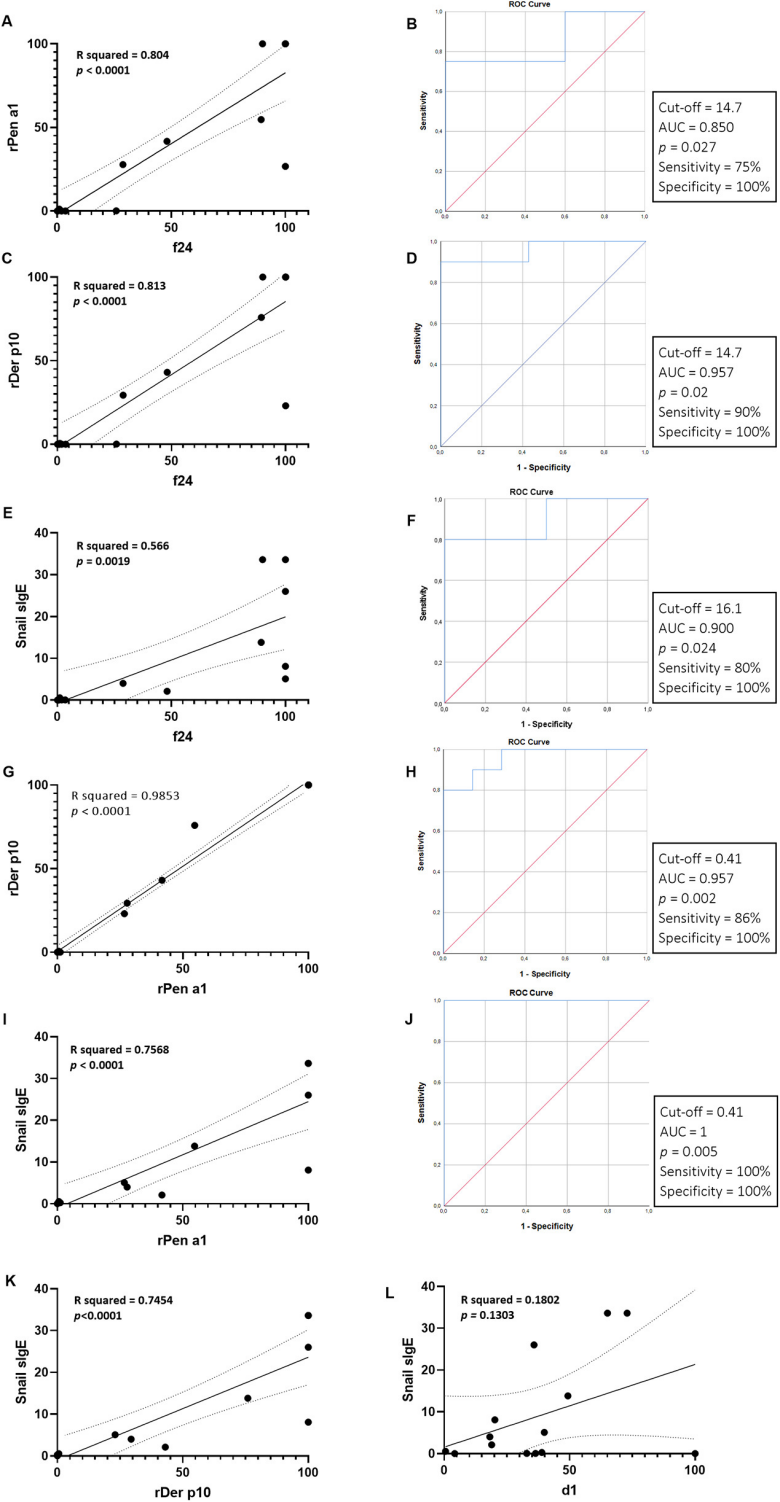
Univariate analysis of predictive factors of sIgE to shrimp: **(A,B)** correlation of molecular diagnosis of shrimp allergy using rPen a1 and whole antigen extract (f24) and receiver operating characteristic (ROC) curve analysis for the diagnostic performance of f24 for rPen a1. **(C,D)** Association between shrimp allergy and sensitization HDMs using rPen a1 and ROC curve analysis for the diagnostic performance of rDer p10 for f24. **(E,F)** Association between shrimp allergy and sensitization to snail and ROC curve analysis for the diagnostic performance of sIgE to snail for f24. **(G,H)** Correlation of molecular diagnosis of shrimp and HDMs allergy using rPen a1 and rDer p10 and ROC curve analysis for the discriminatory ability of rPen a1 to predict rDer p10 sensitization. **(I,J)** Correlation between snail sIgE titers and rPen a1 **(I)**, f24 **(E)**, rDer p10 **(K)** and d1 **(L)** and ROC curve analysis for the discriminatory ability of rPen a1 to predict snail sensitization **(J)**.

The median rPen a1 titer in our study population was 28.42 [5.78–51.05] kUA/L, with 12 patients (70.5%) testing positive. A strong positive correlation was observed between rPen a1 titers and shrimp- sIgE titers, as evidenced by Spearman's rank correlation coefficient (r_s = 0.771, *p* < 0.001) (Figure 4A). ROC curve analysis revealed that a cut-off titer of f24 at 14.7 yielded optimal diagnostic performance for predicting a positive rPen a1 result, with a sensitivity of 75% and specificity of 100% (AUC = 0.850, *p* = 0.027) (Figure 4B). Multiple linear regression analysis revealed significant associations between shrimp sIgE and sIgE to rDer p10 (Figures 4C,D) and snail (Figures 4E,F).

Among the seventeen patients with shrimp-specific IgE positivity (54.8%), all exhibited cross-reactivity and/or symptoms indicative of allergy to at least one additional allergen, including both food and respiratory allergens. While all patients demonstrated cross-reactivity to respiratory allergens, ten individuals (58.8%) also displayed clinical manifestations of cross-reactivity to food allergens. Six (35.2%) patients exhibited associated food allergies without evidence of cross-reactivity. These findings are summarized in Tables 3, 4.

**Table 4:** Description of associated allergies in patients with shrimp-specific IgE positivity.

Associated allergies	Positivity rate	sIgE (kUA/L)
Respiratory allergies		
Cypress	1	0.37
Olive tree	1	3.85
Food allergies		
Fish	2/4	0.95 [0.04–2.30]
Tuna	3/4	2.39 [0.25–5.61]
Milk	1	1.2
Peach (LTP)	2/2	0.61 [0.23–0.99]
Apple	3/3	0.63 ± 0.49
Cashew nut	1	1.93
Almond	1	0.77
Pistachio	1	2.06
Egg white	1/2	0.95 [0.05–1.85]
Sesame	2/2	1.67 [0.16–3.18]
Wheat (rTri a 19 *ω* 5 gliadine)	1	3.37

Univariate analysis examining the association between shrimp-sIgE titers and d1 revealed non-significant correlations, as evidenced by Spearman's rank correlation coefficient (r_s = 0.358, *p* = 0.159). Additionally, the results of our study did not demonstrate a significant association between d1 and rPen a1 (r_s = 0.281, *p* = 0.274). These findings indicate that neither shrimp-sIgE nor rPen a1 are significant predictors of d1 variation. These sera were subsequently analyzed for rDer p10. The median titer for rDer p 10 was 0.56 [5 × 10^−5^–87.95] kUA/L (Figure 4). rDer p10 positivity was observed in 58.8% (10/17) of patients. Univariate analysis demonstrated significant correlations between rDer p10 and sIgE to shrimp (r_s = 0.842, *p* < 10^−3^) and between rDer p10 and rPen a1 (r_s = 0.919, *p* < 10^−3^). A linear regression analysis was conducted to investigate the relationship between rPen a1 and rDer p10. The results indicated a strong positive linear correlation between the two variables, with 98.5% of the variation in Der p10 that could be explained by rPen a1 (R-squared = 0.985) (Figure 4G). The overall significance of the model was confirmed (*p* < 10^−3^). Furthermore, ROC curve analysis identified an optimal rPen a1 cut-off titer of 0.41 for predicting positive rDer p10 results, yielding a sensitivity of 86% and specificity of 100% (AUC = 0.957, *p* = 0.002) (Figure 4H). Screening for sIgE to snail was conducted among the 17 individuals who tested positive for shrimp sIgE. The median titer of for sIgE to snail was 3.07 [0.04–16.85] kUA/L (Figure 3). Of the 17 patients, 10 (58.8%) exhibited positive sIgE responses to snail antigens.

The results of our investigation demonstrated significant correlations between sIgE to snail and sIgE to shrimp (r_s = 0.877, *p* < 10^−3^) and between sIgE to snail and rPen a1 (r_s = 0.920, *p* < 10^−3^). While no significant correlation was identified between sIgE to snail and d1 (r_s = 0.425, *p* = 0.130), a statistically significant association was found between sIgE to snail and rDer p10 as evidenced by Spearman's rank correlation coefficient (r_s = 0.912, *p* < 10^−3^). Multiple linear regression analyses conducted on sIgE levels to snail revealed strong, positive linear correlations with shrimp, rDer p10 and rPen a1. These findings suggest that individuals with elevated sIgE levels to snail are more likely to exhibit heightened sensitivity to these related allergens. The R-squared values, ranging from 0.566 to 0.757, indicate that a substantial portion of the variation in sIgE to snail can be explained by the sensitization to shrimp, rDer p10 and rPen a1. Moreover, the significant *F*-tests confirm the statistical significance of the models, providing strong evidence for the existence of these relationships (Figures 4I,J). Additionally, a highly accurate prediction of sensitization to snails was achieved using a cut-off titer of rPen a1 of 0.41, yielding 100% sensitivity and 100% specificity (AUC = 1, *p* = 0.005) (Figure 4J).

Multivariable analysis of predictive factors of shrimp sIgE (f24) demonstrated a significant association with rPen a1 titer and sensitization to rDer p10 (*p* = 0.003, *p* < 10^−3^, respectively). No correlation was observed between shrimp sIgE, d1 and snail sIgE levels (*p* = 0.689, *p* = 0.080, respectively) (Table 5).

**Table 5:** Associated factors to f24 by multivariable analysis after adjusting for age.

Final model	Beta coefficient	*T* test	Sig.	CI 95% beta coefficient	
				Inf	Sup
Age	0.017	0.688	0.51741	−0.100	0.179
rPena1	−0.674	−4.674	0.00342	−1.050	−0.328
d1	−0.010	−0.421	0.68855	−0.112	0.079
rDerp10	1.741	12.212	0.00002	1.379	2.070
Snail	−0.084	−2.102	0.08026	−0.704	0.053

Dependent variable: f24.

Multivariable analysis of predictive factors of snail sensitization showed no statically significant associations with sIgE to shrimp, to rPen a1 and to d1 and to rDer p10. The model explained approximately 77.8% of the variation of sIgE to snail. Durbin-Watson statistic of 1.36 suggests no significant autocorrelation in the residuals. Among the independent variables, rDer p10 and f24 were the strongest predictors of sIgE to snail, with standardized coefficients of 8.785 and −5.028, respectively, and partial correlation coefficients of 0.302 and −0.311, respectively. However, these associations were not statistically significant (*p* = 0.087 and *p* = 0.080, respectively) (Table 6).

**Table 6:** Associated factors to sIgE positivity to snail by multivariable analysis after adjusting for age.

Final model	Beta coefficient	*T* test	*P*	CI 95% beta coefficient		Partial correlation
				Inf	Sup	
Age	0.018	0.094	0.928	−0.279	0.301	0.014
rPena1	−2.976	−1.438	0.200	−2.129	0.553	−0.212
d1	0.096	0.517	0.624	−0.149	0.229	0.076
rDerp10	8.785	2.045	0.087	−0.443	4.953	0.302
f24	−5.028	−2.102	0.080	−2.819	0.214	−0.311

Dependent variable: snail sIgE.

## 4 Discussion

This study constitutes the pioneering comprehensive investigation into the phenotypic profile of shrimp allergy in a Tunisian population. In our population, an association between this food allergy and snails has been observed, accompanied by a wide range of adverse food reactions. This highlights the need for region-specific epidemiological data on food allergens. The prevalence of seafood allergies tends to be higher in populations when consumption plays a greater part in the diet of the observed community (16, 17). Sensitization to shrimp allergens occurs most frequently through ingestion but also via dermal contact, or inhalation of aerosolized proteins generated during food preparation or processing (18). Diagnosis of shrimp allergy relies on clinical history, skin prick tests (SPTs) with hole or commercial extract allergens, serum specific IgE and specifically component-resolved diagnosis. Whenever feasible and safe, confirmation is obtained through an oral food challenge (OFC). In our study group, no OFC were conducted due to their high risk of anaphylaxis. Shrimp allergy, with a positivity rate of 54.8% (17/31) in the study cohort, is emerging as a notable public health concern, influenced by evolving dietary patterns. To date, no studies have investigated the prevalence of shrimp allergy in Tunisia, despite indications of a rising trend, potentially fueled by the increasing popularity of Asian cuisine and cultural influences. In alignment with existing literature which have consistently shown that shellfish allergy is often diagnosed later in life, with peak onset during adolescence (19), our findings demonstrate that shellfish allergy is frequently diagnosed at a later age, as the mean age of shrimp-allergic patients was 16 [12–28] years. A male predominance, with a gender ratio (male/female) of 1.8, was observed in our study, aligning with findings from most pediatric studies (20), whereas food allergies in adults are typically more prevalent among females (21). Adverse reactions to shrimp encompass a spectrum of conditions, including immunological, toxic, and non-immunological responses (22). While food protein-induced enterocolitis syndrome (FPIES) and toxic reactions are significant, this article will focus on IgE-mediated allergic reactions amenable to biological exploration and the most allergenic protein involved. The clinical presentation of shrimp allergy is often characterized by a rapid onset of symptoms within two hours of exposure (23), aligning with the typical profile of immediate-type food allergies. However, delayed phase reactions extending up to eight hours post-ingestion have also been reported (24). Crustaceans and mollusks are recognized as primary triggers of severe, potentially life-threatening, immediate-type food allergic reactions, including anaphylaxis and food-dependent exercise-induced anaphylaxis (FDEIA) (16, 25–27). The severity of allergic reactions to shrimp can exhibit significant variability even within the same individual, influenced by factors such as food preparation methods, specific shrimp species, and the presence of potential co-factors, including alcohol, exercise, and nonsteroidal anti-inflammatory drugs (*N*SAIDs) (28–30). The clinical spectrum of shrimp allergy in our study population was heterogeneous. While most patients (82.3%) were presented with mild to moderate symptoms, three patients (17.6%) experienced severe reactions characterized by rapid edema. In two cases (17.6%), these reactions escalated to life-threatening anaphylaxis necessitating intensive care unit intervention. Our findings align with the existing literature, where the reported prevalence of anaphylaxis varies across studies, ranging from 8.3% to 33% (2, 26). Respiratory manifestations and oral allergy syndrome are more commonly associated with shellfish allergies compared to other food allergies (2). Perioral symptoms constituted the predominant clinical manifestation, affecting 64.7% (11/17) of patients. Severe shrimp aversion was reported by 17.6% (3/17) of patients and tropomyosin-specific IgE was positive in two of them. Cutaneous manifestations, including urticaria and pruritus, were reported in 29.4% (5/17) of participants. These cutaneous reactions were consistently associated with respiratory symptoms. In two cases, severe reactions, such as rapid edema, followed the onset of these cutaneous manifestations. Gastrointestinal symptoms were documented in two patients, with one of them exhibiting isolated eosinophilic esophagitis in the absence of systemic involvement. In contrast to many other food allergies, shellfish allergy often follows a persistent course, affecting up to 90% of patients throughout their lifetime, akin to the pattern observed in peanut allergy (1). This long-term persistence is corroborated by findings from our study group, where follow-up data and patient clinical histories revealed a chronic course in all documented cases. The measurement of total IgE (tIgE) is not recommended as a primary diagnostic tool for allergy, as its levels can be influenced by various conditions, including parasitic infections and inflammatory diseases, limiting its specificity. However, tIgE can serve as a potential indicator of atopy, and the clinical relevance of sIgE levels may depend on tIgE levels (31). The added value of considering tIgE and sIgE/tIgE ratios in improving the diagnostic accuracy of sIgE measurement remains debated (32). In this study, tIgE levels, as well as the sIgE/tIgE and rPen a1/tIgE ratios, were significantly correlated with f24, rPen a1, and rDer p10. Similar findings have been reported by other authors regarding the predictive value of tIgE in food allergies (31, 33). Although the sIgE/tIgE ratio did not correlate with symptom severity in our study population, previous research suggests that this ratio is more accurate than sIgE alone in predicting oral food challenge (OFC) outcomes. It has been proposed as a useful serological marker for identifying patients more likely to pass OFCs and develop tolerance to specific food allergens, as well as for guiding the initiation of specific immunotherapy (33–35). The lack of statistical significance observed in our study may be attributed to the small sample size. Moreover, food allergy is generally recognized as a risk factor for the development of asthma with an odds ratio of 2.16 (36), underscoring a potential shared atopic predisposition or cross-reactive epitopes. Roberts et al. reported a six-fold increased risk of severe asthma in children with early-onset food allergies, supporting the atopic march (37, 38). Similarly, Wang et al. found higher rates of asthma, allergic rhinitis, and familial atopy in children with shellfish allergies (39). In accordance with these findings, our study demonstrated a high prevalence of atopic symptoms, such as allergic asthma, urticaria and allergic rhinitis in patients sensitized to shrimp. Specifically, two (11.7%) shrimp-allergic patients had asthma, five (29.4%) experienced urticaria, and ten (58.8.%) suffered from allergic rhinitis. Despite the absence of reported allergic rhinitis in seven patients, comprehensive serological analysis revealed the presence of sIgE antibodies against the HDMs allergen d1 in all participants. The observed lack of significant associations between d1 and both shrimp sIgE and rPen a1 might be due to the small sample size and the presence of confounding factors. Conversely, allergic multimorbidity, particularly the coexistence of shellfish and respiratory allergies, appears to heighten the risk of anaphylaxis, especially in humid environments (40). In our series, three cases of anaphylaxis were documented. The first case involved a 73-year-old male physician with a history of allergic rhinitis and chronic urticaria, who experienced rapid edema following shrimp ingestion. This reaction triggered a myocardial infarction, requiring immediate admission to the intensive care unit. Serological investigations revealed sensitization to shrimp (f24 = 3.48 kUA/L), crab (f23 = 1.37 kUA/L), and HDMs (d1 = 36.4 kUA/L), while specific IgE to rPen a 1 and rDer p 10 were negative, suggesting unrecognized sensitization to HDMs. The second case involved a 17-year-old female with a history of allergic asthma and pollinosis, who developed severe anaphylaxis after consuming a single shrimp. She required intensive care admission. Serological testing demonstrated sensitization to shrimp (f24 = 89.4 kUA/L, rPen a1 = 54.7 kUA/L), crab (f23 = 98.2 kUA/L), snail (f314 = 13.8 kUA/L), sesame, and apple (f49 = 0.52 kUA/L), with no evidence of fish sensitization (f3 = 0.07 kUA/L). Elevated HDMs sIgE levels (d1 = 49.3 kUA/L and rDer p10 = 75.9 kUA/L) confirmed HDMs sensitization. The third case involved a 15-year-old male who developed severe grade III anaphylaxis after consuming shrimp, requiring intensive care admission. Serological analysis confirmed shrimp sensitization, with markedly elevated sIgE levels (f24 > 100 kUA/L, rPen a1 > 100 kUA/L), along with increased sensitization to house dust mites (HDMs) (d1 = 20.2 kUA/L, rDer p10 > 100 kUA/L) and snail (f314 = 8.09 kUA/L). Notably, all three patients exhibited rapid-onset edema and had a history of allergic rhinitis. Their serological profiles consistently indicated HDM sensitization, with a mean d1 titer of 61.9 ± 33.6 kUA/L. These findings underscore the severe and potentially life-threatening nature of shrimp allergy, often linked to a broader allergic profile that includes sensitization to both food allergens and inhalants, particularly HDMs. Our results are consistent with previous studies showing an association between HDMs sensitization and subsequent shellfish sensitization, likely due to cross-reactivity between tropomyosins or other heat-stable proteins in crustaceans and HDMs (41). In this study, sensitization to shellfish tropomyosins was assessed through specific serum IgE measurement using the ImmunoCAP® assay, which offers superior sensitivity and specificity for predicting shellfish allergy compared to skin prick tests with whole shrimp extract (42). Tropomyosin, a major allergen in both HDMs (rDer p10) and shrimp (rPen a1), is a well-characterized pan-allergen with high structural conservation. It shares a notable degree of amino acid sequence homology (78.5–81.7%) (40), contributing significantly to cross-reactivity among diverse invertebrates, including HDMs, cockroaches, crustaceans, and mollusks (12). Given the high consumption of snails in our country, we investigated snail allergy and identified a positivity rate of 58.8%. The strong correlation observed between sIgE to snail, shrimp, rPen a1, and rDer p10, combined with the high frequency of snail allergy among individuals sensitized to shrimp, supports the hypothesis of a shared immunological basis for these allergens. In our study, 70.5% of patients tested positive for rPen a1, and 58.8% were sensitized to rDer p10, highlighting tropomyosin as a key allergen among Tunisian individuals. A strong correlation was observed between rPen a1, rDer p10, and snail allergens. Predictive modeling further supported these findings, with a shared cut-off value of 0.41 kUA/L for predicting sensitization to both HDMs (rDer p10) and snails. Consistent with our results, a study by Sanchez et al. demonstrated that an rDer p10 IgE level greater than 1.2 kUA/ml exhibited optimal diagnostic performance, with 100% sensitivity and 65% specificity, for predicting shrimp allergy among patients with allergic rhinitis (43). These findings emphasize the importance of accurate diagnostic tools, such as Component-Resolved Diagnosis (CRD), to identify tropomyosin sensitization and guide education for at-risk individuals. Patients sensitized to tropomyosin (rPen a1) exhibited clinical manifestations ranging from mild symptoms to severe anaphylaxis following shrimp ingestion. Notably, two patients experienced severe allergic reactions, including rapid-onset edema, despite negative sIgE to rPen a1. Both were sensitized to HDMs; however, molecular analysis revealed negative rDer p10 results. Additionally, testing for snail allergy was negative. These IgE-mediated reactions suggest potential sensitization to other shrimp allergens, such as Pen m 1, or to non-tropomyosin components like arginine kinase (Pen m2) and the heat-stable sarcoplasmic calcium-binding protein (Pen m4). These allergens, which are available for testing via the ISAC microarray platform (Thermo Fisher Scientific™), are currently unavailable in our diagnostic panel. The reliance on a single allergen, such as rPen a1, for IgE detection may underestimate sensitization, particularly in patients with clinical symptoms but negative rPen a1 results. This underscores the need for broader allergen panels to enhance diagnostic accuracy and better characterize sensitization profiles, especially in patients with complex allergic presentations. In our series, we also observed cases of patients with negative rDer p10 and positive d1, indicating sensitization to HDMs without clinical manifestations of HDMs allergy. One notable patient exhibited a distinctive profile, characterized by high d1 levels (d1 > 100 kUA/L), low but detectable sIgE to rPen a1 (rPen a1 = 0.22 kUA/L), and a history of wheat-dependent exercise-induced anaphylaxis. This patient had suffered for over a decade from allergic asthma and allergic rhinoconjunctivitis consistent with house dust mite allergy. Despite these findings, rDer p10 sensitization was absent, suggesting potential sensitization to a HDMs allergen other than rDer p10. Although tropomyosin is a well-established cross-reactive allergen, it is important to recognize that other shellfish allergens, such as arginine kinase, hemocyanin, and myosin heavy chain type 1, may contribute to immunological cross-reactivity between shellfish and inhalant allergens (44–46). These findings underline the necessity of a comprehensive diagnostic approach, including component-resolved diagnostics, to identify specific allergens and develop clear, unified management strategies. Our study has some limitations. Its retrospective design and relatively small sample size precluded a thorough evaluation of long-term outcomes, such as the progression to asthma or rhinitis in patients with shrimp allergy, whether sensitized to HDMs or not. To better understand the complex interplay between shrimp allergy, HDMs sensitization, and allergic comorbidities, prospective studies with larger sample sizes are warranted.

## 5 Conclusion

This study underscores the growing emergence of shrimp allergy in the Tunisian population and its strong association with sensitization to HDMs and snails. The true prevalence of shrimp allergy is likely underestimated due to limited access to biological diagnostic tools for many Tunisian patients. Further research is essential to identify factors contributing to its occurrence. To date, no studies have systematically evaluated shrimp allergy prevalence in Tunisia. To address this gap, a large-scale investigation incorporating mass screening is imperative to achieve a comprehensive understanding of this condition. A standardized, self-reported questionnaire modeled on the Food Allergy Questionnaire from the Royal Children's Hospital in Melbourne, Australia, should be administered to a representative population sample. This effort should be complemented by objective diagnostic methods, such as double-blind, placebo-controlled food challenges, to capture cases beyond those typically reported through clinical settings. Such an approach would provide valuable insights for developing effective prevention and management strategies. Given the central role of tropomyosin as a major allergen in shrimp allergy within the Tunisian population, further studies on tropomyosin sensitization are warranted to confirm and expand upon our findings. These studies could elucidate the mechanisms underlying cross-reactivity between tropomyosins from different species and other allergens. Clinically, educating patients on avoiding shrimp and cross-reactive inhalant allergens could help mitigate the risk of further sensitization and IgE-mediated allergic reactions.

## Data Availability

The original contributions presented in the study are included in the article/Supplementary Material, further inquiries can be directed to the corresponding author.
